# Dynamic pH Sensor with Embedded Calibration Scheme by Advanced CMOS FinFET Technology

**DOI:** 10.3390/s19071585

**Published:** 2019-04-02

**Authors:** Chien-Ping Wang, Ying-Chun Shen, Peng-Chun Liou, Yu-Lun Chueh, Yue-Der Chih, Jonathan Chang, Chrong-Jung Lin, Ya-Chin King

**Affiliations:** 1 Institute of Electronics Engineering, National Tsing Hua University, Hsinchu 30013, Taiwan; jpwang.starlab@gmail.com (C.-P.W.); pjliou.starlab@gmail.com (P.-C.L.); cjlin@ee.nthu.edu.tw (C.-J.L.); 2 Institute of Materials Science and Engineering, National Tsing Hua University, Hsinchu 30013, Taiwan; kuroekyo@gmail.com (Y.-C.S.); ylchueh@mx.nthu.edu.tw (Y.-L.C.); 3 Design Technology Division, Taiwan Semiconductor Manufacturing Company, Hsinchu 30075, Taiwan; ydchih@tsmc.com (Y.-D.C.); jon_chang@tsmc.com (J.C.)

**Keywords:** pH sensors, readout circuit scheme, calibration operation, CMOS FinFET technology

## Abstract

In this work, we present a novel pH sensor using efficient laterally coupled structure enabled by Complementary Metal-Oxide Semiconductor (CMOS) Fin Field-Effect Transistor (FinFET) processes. This new sensor features adjustable sensitivity, wide sensing range, multi-pad sensing capability and compatibility to advanced CMOS technologies. With a self-balanced readout scheme and proposed corresponding circuit, the proposed sensor is found to be easily embedded into integrated circuits (ICs) and expanded into sensors array. To ensure the robustness of this new device, the transient response and noise analysis are performed. In addition, an embedded calibration operation scheme is implemented to prevent the proposed sensing device from the background offset from process variation, providing reliable and stable sensing results.

## 1. Introduction

Providing real-time and accurate levels of ion concentration through sensing modules in smart electronic devices can be of high interest to the development of many new applications [1,2]. For instance, in personal health monitoring devices [2], detecting the pH level in the blood samples can be applied to offer real-time diagnosis of many diseases like acidosis and alkalosis [3]. In addition, it can be useful in clinical care, where real-time monitoring of changes in ion levels enables early detection of the on-set of critical conditions in patients [4]. Other than the above, in agriculture, feedback from pH detectors in Internet of Things (IoT) devices planted in soil can also be of great potential for the maintenance of optimal crop productivity across large areas of fields [5]. Therefore, ion sensors which can be embedded into smart and/or IoT devices can play an important role in medical care, agriculture as well as environmental monitoring systems over wide ranges. 

The development of discrete electronic ion sensors has been proposed and implemented by many different technologies [6,7]. For better compatibility to CMOS processes, field effect transistor (FET)-based solutions are considered most feasible. Ion-sensitive field effect transistor (ISFET) was the first FET-based ion sensor which was introduced by Piet Bergveld in 1970 [8]. The first ISFET was designed without a gate electrode, while the corresponding channel regions, isolated only by a thin dielectric film to the electrolyte solution, are then controlled by the electron potential generated at the coated surfaces [9]. According to the site-binding model [10], the hydrogen ions in the electrolyte solution will react with the coating layer as described by the following equations:

(1) $${\mathrm{MH}}_{2}^{+}⇌\mathrm{MH}+H^{+},$$

(2) $$\mathrm{MH}⇌M^{-}+H^{+},$$

where ${\mathrm{MH}}_{2}^{+}$, MH, $M^{-}$ represent positive, neutral and negative surface sites, respectively.

The chemical reaction at the coated surfaces changes the interface potential, which in turn affects the subsequent FET current level [10,11]. At equilibrium, electrolyte samples with different pH levels, namely, H^+^ ions reacting to the coating film, will induce a change in the surface potential, leading to a shift in channel current during a fixed read condition, responsively [11,12]. Based on fundamental electrochemistry rules, the relation between ion concentration and the interface potential can be predicted by the following equations:

(3) $$\Psi_{0}=E_{0}-\frac{2.303\mathrm{RT}}{F}\mathrm{pH},$$

(4) $$\Delta \Psi_{0}=\frac{2.303\mathrm{RT}}{F}\Delta \mathrm{pH},$$

(5) $$\frac{\Delta \Psi_{0}}{\Delta \mathrm{pH}}=\frac{2.303\mathrm{RT}}{F}=59.6\;(\mathrm{mV}/\mathrm{pH}),$$

where $\Psi_{0}$ is the interface potential, and R and F stand for the gas and Faraday constant, respectively. As calculated from Equation (5), the maximum sensitivity will be 59.6 mV/pH, which is also known as the Nernst limit [13]. 

The extended-gate ion-sensitive field effect transistor (EGFET) was proposed by J. Van der Spiegel in 1983 [14]. Unlike ISFET, the ion sensing layer is coated to the extended gate, which is connected to the gate of the sensing FET through internal wiring. The electrolyte solution can then be isolated from the transistor channel region, consequently, the deposition of ion-sensitive coating can be done with much more flexibility through post CMOS processes. This not only prevents contamination concerns in the exposed gate structure, but also allows for more options which optimize the sensitivities of the coating layer [15,16]. Moreover, EGFET can be fully assembled with integrated readout circuits, therefore, the interferences from the surroundings, i.e., light, heat or other noise sources, can be effectively minimized [17]. 

However, the reading from both ISFET and EGFET are easily disturbed by the noise in the environment [18]. Additionally, their pH sensing ranges are fixed, determined by the material of the corresponding sensing layers [19]. Without a well-controlled gate biased, the read current level as well as sensitivity of these conventional ISFET are hard to predict and subjected strongly to process variations as well as the testing environment [20]. A novel ion detector with self-balanced readout circuit scheme which features high and flexible sensitivity, adjustable sensing range and fully COMS FinFET process compatible was proposed in our previous work [21]. In this paper, we further explore the characteristics of this device with more comprehensive discussion in its performance optimization, speed and operation limitations.

## 2. Device Characteristic and Experimental Section

### 2.1. Device Structure and Operation Principle

**Figure 1:**
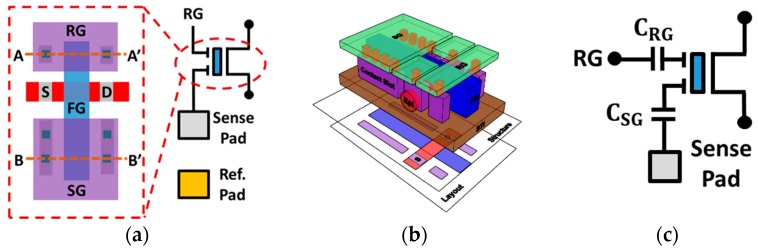
(**a**) The circuit schematic and corresponding layout of the proposed ion detector with the extended floating metal gate coupled by slot contacts on both sides. (**b**) The 3D illustration of the proposed ion sensor. (**c**) The capacitance model of sense gate (SG) and read gate (RG) in the novel ion detector.

Figure 1a shows the schematic and layout of the proposed Fin Field-Effect Transistor (FinFET) process compatible ion sensor studied in this work. In addition, the 3D structure is illustrated in Figure 1b. This device consists of a n-channel Metal-Oxide-Semiconductor Field-Effect Transistor (MOSFET) with floating gate (FG) coupled by two input gates, sense gate (SG) and read gate (RG), respectively. Since this metal gate on top of the fin-shaped channel is floated, its potential is controlled by the amount of charge it stored and the potential of two external terminals, SG and RG. The potential of both input gates can be capacitively coupled to the common floating metal gate (FG) through a lateral structure on the top of the shallow trench isolation (STI), and subsequently, the channel current of the FinFET will be affected. The capacitance model of SG and RG is shown in Figure 1c.

**Figure 2:**
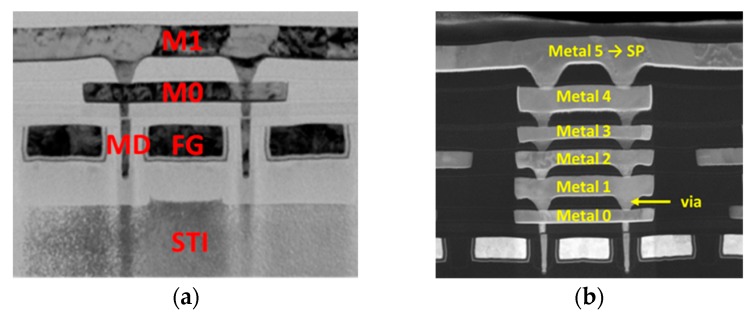
Transmission Electron Microscope (TEM) picture along (**a**) AA’ and (**b**) BB’ cut-lines shown in Figure 1, respectively, showing how the lateral coupling structures are fabricated under standard Fin Field-Effect Transistor (FinFET) processes.

To further demonstrate the coupling structure, the Transmission Electron Microscope (TEM) picture along AA’ cutline is shown in Figure 2a. Two slot contacts are deliberated placed close to both sides of the floating metal gate, forming two long-stripe metal-insulator-metal (MIM) lateral capacitors between the slot contacts and FG. As a result of the high aspect ratio metal gate as well as the narrow gap between the contact and metal gate enabled by advanced FinFET processes, an area efficient laterally coupling structure can be obtained. The unit length capacitance of this lateral structure can reach as high as 0.134 fF/μm in 16 nm FinFET processes. With continue scaling in lateral dimensions, this coupling effect is expected to be more enhanced in future technology nodes beyond 16 nm.

By the help of the coupling capacitors, RG is designed to exert stronger control on the sensing current, while SG, which is connected to the sense pad (SP) through multiple metal and via layers as the TEM picture along BB’ line, as shown in Figure 2b. SG transmit the potential change detected in SP affected by the ion concentration of the electrolyte solution. 

### 2.2. Operation Principle

**Figure 3:**
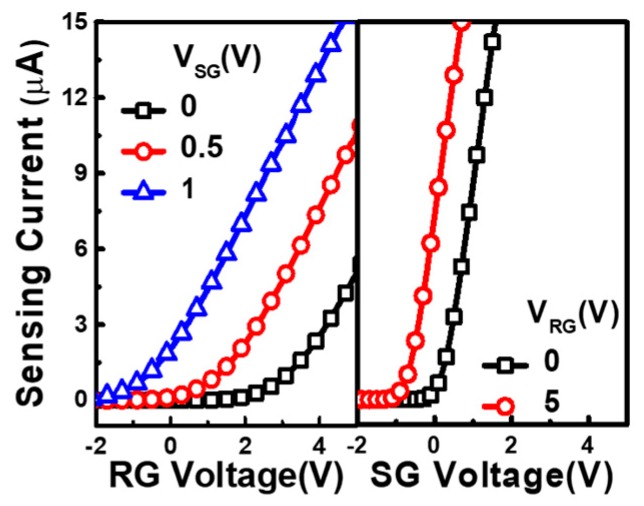
Transfer characteristics of the proposed ion sensor in response to different given SG and RG voltage, respectively. As shown, SG is designed to have stronger influence to the sensing current.

The measured transfer characteristics of the ion detector are compared in Figure 3, when either one of the input gates is swept with the other gate under a fixed bias. As the data revealed, the potential difference on the SG can directly affect the I-V curve under fixed RG voltage ($V_{\mathrm{RG}}$). Similarly, the sensing current characteristics when one sweeps $V_{\mathrm{RG}}$ can shift when different SG voltage ($V_{\mathrm{SG}}$) is given.

The FG potential is mainly controlled by the coupling capacitances from SG and RG to FG, respectively. To further calculate the FG potential, the coupling ratio (α) from each gate is defined as follows [22]:

(6) $$\alpha_{\mathrm{SG}}=\frac{C_{\mathrm{SG}-\mathrm{FG}}}{C_{\mathrm{FG},\mathrm{total}}};{\;\alpha }_{\mathrm{RG}}=\frac{C_{\mathrm{RG}-\mathrm{FG}}}{C_{\mathrm{FG},\mathrm{total}}},$$

where $C_{\mathrm{SG}-\mathrm{FG}}$ and $C_{\mathrm{RG}-\mathrm{FG}}$ is the capacitance from FG to SG and RG, respectively, while $C_{\mathrm{FG},\mathrm{total}}$ is the total capacitance of FG, which is defined as follows:

(7) $$C_{\mathrm{FG},\mathrm{total}}=C_{\mathrm{SG}-\mathrm{FG}}+C_{\mathrm{RG}-\mathrm{FG}}+C_{S-\mathrm{FG}}+C_{D-\mathrm{FG}}+C_{B-\mathrm{FG}},$$

where $C_{S-\mathrm{FG}}$, $C_{D-\mathrm{FG}}$ and $C_{B-\mathrm{FG}}$ represent the capacitance between source/drain/substrate and floating gate.

The definition of subthreshold swing (S.S.) is the change in applied bias required to change the subthreshold drain current ($I_{D}$) by one decade [23]. S.S. can be obtained based on measured I-V curves using the following equation.

(8) $$S.S.\equiv \;\frac{1}{\partial {\mathrm{logI}}_{D}/\partial V_{g}}.$$

Since S.S. represents the gate control capability of one device, the coupling ratios of FG from SG and RG can be obtained by finding the S.S. when gate sweep is changed to SG and RG, respectively. As shown in the following equation, the corresponding coupling ratios can be calculated based on measured IV data [24].

(9) $$\alpha_{\mathrm{SG}}=\frac{S.S._{\mathrm{dummy}}}{S.S._{\mathrm{SG}}};\;\alpha_{\mathrm{RG}}=\frac{S.S._{\mathrm{dummy}}}{S.S._{\mathrm{RG}}},$$

where $S.S._{\mathrm{dummy}}$ represent the subthreshold swing of the dummy device, where the FG is connected to the external gate terminals. $S.S._{\mathrm{SG}}$ and $S.S._{\mathrm{RG}}$ are the subthreshold swings of the floating gate device while sweeping $V_{\mathrm{SG}}$ and $V_{\mathrm{RG}}$, respectively.

Through mathematical calculation, Equation (6) is proved to match the measured result of Equation (9). Therefore, the coupling ratio of the corresponding gate can be extracted from the sub-threshold I-V curve, which make it much easier to be obtained and estimated.

**Figure 4:**
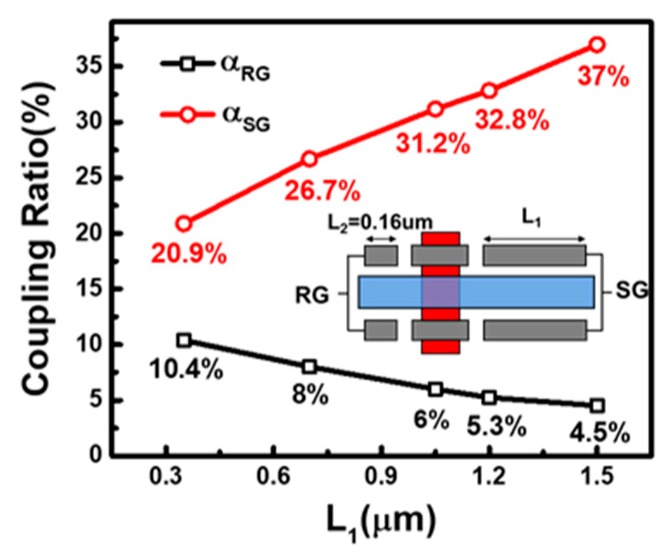
Relationship between the coupling ratios from SG/RG, calculated from measured sub-threshold characteristics, in response to the increase of the corresponding slot contact length.

The lateral capacitors are formed between contact slots and the FG, hence, the length of contact is directly in proportional to the coupling capacitance, which make it easily adjustable by a simple change in the device layout. As revealed by the measured data in Figure 4, for samples with fixed length on the RG ($L_{2}$) of 160 nm, increasing the length of SG ($L_{1}$) will linearly increase the coupling ratio from the SG, as expected. Namely, the coupling ratio can be precisely controlled and optimized by adjusting the length of contact. On the other hand, as $L_{1}$ increases, the total FG capacitance raises, hence, the coupling ratio from RG decreases as expected (see Figure 4). The linear dependence between $\alpha_{\mathrm{SG}}$, $\alpha_{\mathrm{RG}}$, $L_{1}$ and $L_{2}$ further suggests that the coupling structure dominantly controls the FG potential, which is beneficial for precise design of the characteristics of the device to meet optimal needs for the specific sensing applications. 

### 2.3. Experimental Setup

**Figure 5:**
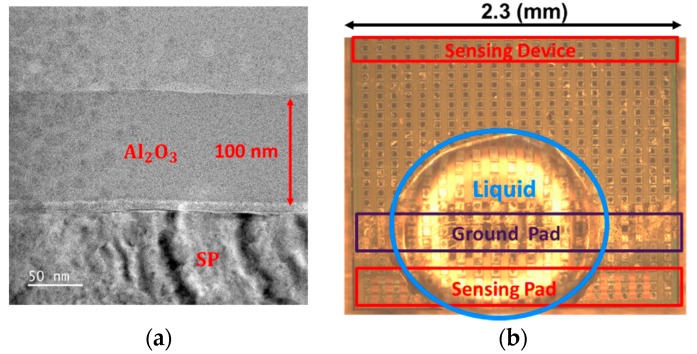
(**a**) The TEM image of the cross-section view of the sensing region. (**b**) Photograph showing the pH sensing experimental setup with post CMOS coating on the sensing pad and the ground electrode.

After the main devices are fabricated by standard CMOS FinFET processes, Argon plasma is used to remove the passivation layer on the top of SP. As demonstrated in the TEM picture in the previous section, the sensing gate of the read transistor is connected to the SP by the metal and via layers by CMOS back-end-of-line (BEOL) process. Once the sensing pad is clear, a layer of aluminum oxide is coated on the top of wafer as the ion sensitive medium with the help of the electron beam evaporation. After the chamber is in vacuum, a high energy electron beam will hit the target material. The atom of target material will then be converted to gaseous state and form a coated layer on the samples. During this process, the chamber temperature will be heated up to about 50–100 °C, which will not affect the circuit underneath. In Figure 5a, the TEM image of the sensing region of the sliced sample obtained by the focus ion beam (FIB) system shows that the thickness of the ion sensitive film coated on top of SP is about 100 nm. Meanwhile, a gold electrode is also added on top of the sample to ensure the ground potential on the electrolyte solution is accurately set. The placement of liquid samples on the sensors after the formation of SP and the ground electrode by post-CMOS processes are shown by the picture in Figure 5b. During pH sensing experiments, the liquid electrolyte solution needs to cover both the SP and the ground electrode to ensure the proper setting of the liquid to ground potential of the circuit system, so that the shift in surface potential can be precisely detected by the underneath sensor. 

## 3. Results and Discussion

### 3.1. Measured Result and Readout Circuit Characteristics

**Figure 6:**
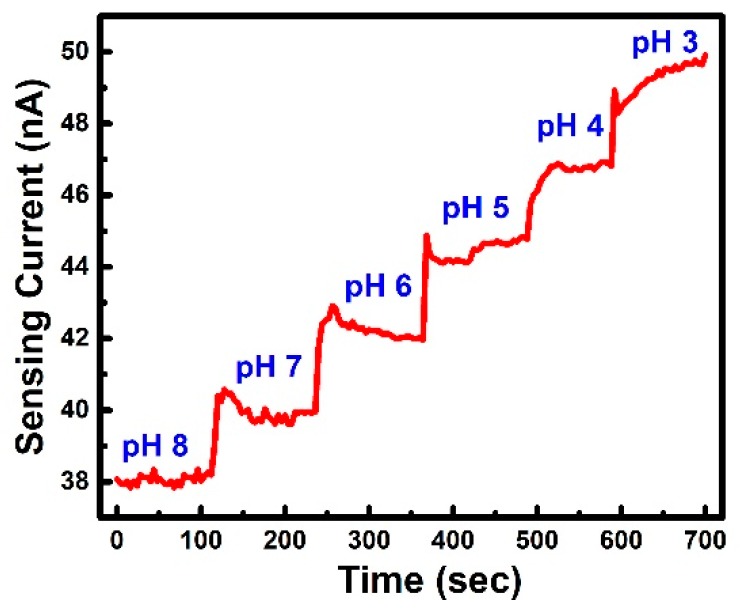
Real-time sensing current in response to the change in test samples with decreasing pH levels where the device is kept at around threshold to ensure high sensitivities.

The real-time pH sensing results under fixed RG bias of 4 V (near threshold voltage) is shown in Figure 6. During the measurement, every pH level of the solution is changed every 100 s. The buffer solution here is KOH/HCl. The volume of solution is 1 μL which is controlled by a pipette and then wiped after measurement. As the measured data revealed, the sensing current under the fixed bias increased as the hydrogen ion concentration decreased, i.e., the pH level increased. Once the equilibrium is reached, the current fluctuation is expected to come from noise in the system. Using the measured noise in sensing current, the signal-to-noise ratio (SNR) can be calculated based on the following equation [25,26]:

(10) $$\mathrm{SNR}=\;\frac{\Delta I_{D}}{i_{D}\left(\mathrm{RMS}\right)}=\frac{\Delta I_{D}}{\sqrt{\int_{f_{1}}^{f_{2}}S_{I}\left(f\right)\mathrm{df}}},$$

where $\Delta I_{D}$ is the signal when the pH levels change from one level to the other and $i_{D}\left(\mathrm{RMS}\right)$ can be accounted for the noise.

The estimated SNR, based on the average change in sensing current per pH, is found to be around 30 dB, when the transistor is operated around threshold region.

**Figure 7:**
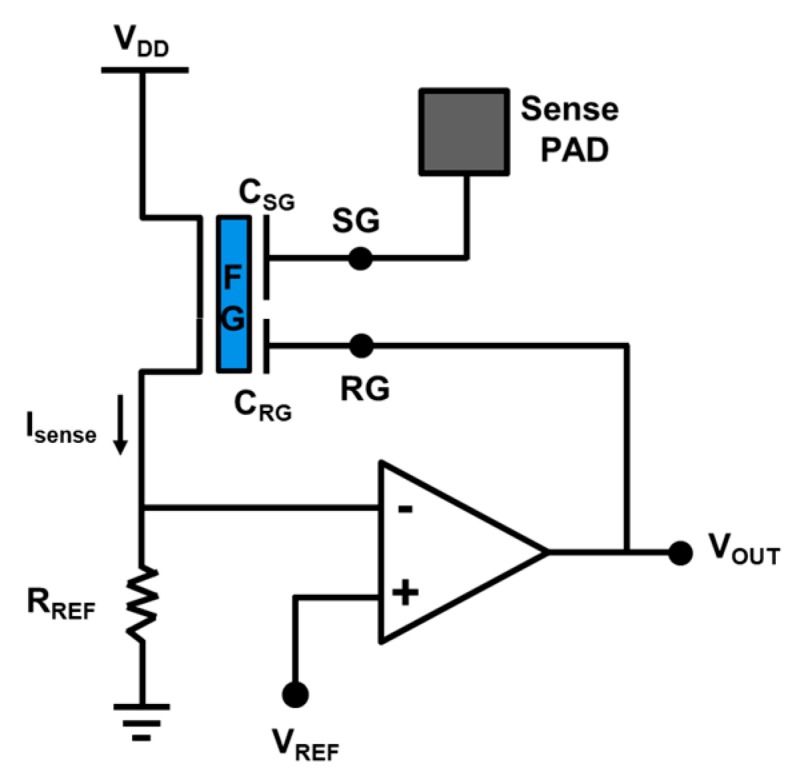
The proposed self-balanced readout circuit scheme for the new ion detector, including an operational amplifier to enable the forming of a strong negative feedback loop for the reading of the sense pad voltage.

The proposed novel self-balanced readout scheme and its corresponding circuit is illustrated in Figure 7. Through a negative feedback loop controlled by an operational amplifier, the $I_{\mathrm{sense}}$ can be set by designing the reference resistor, $R_{\mathrm{REF}}$ as well as the reference voltage, $V_{\mathrm{REF}}$. 

Once the reference voltage $V_{\mathrm{REF}}$ and reference resistor $R_{\mathrm{REF}}$ are set, the sense current $I_{\mathrm{sense}}$ will be fixed if the circuit reaches equilibrium. That is to say that the floating gate potential is forced to be kept at the fixed potential at steady-state. Therefore, when the sense gate voltage increases, the sense current will also increase, which drives the operational amplifier to a lower output voltage $V_{\mathrm{OUT}}$. This lower $V_{\mathrm{OUT}}$ is then fed back to RG which is coupled to the FG to pull the potential down. Once the negative feedback loop is in balance, the output voltage will be inversely proportional to SP voltage $V_{\mathrm{SP}}$, reflecting the corresponding pH level of the liquid sample.

**Figure 8:**
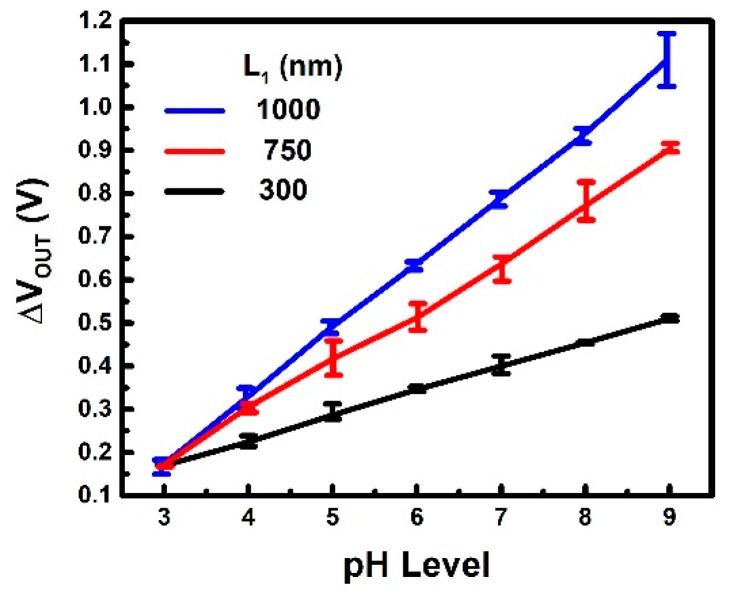
pH measurement results showing linear output response with increasing pH levels of the liquid samples. Responsivities can be enhanced as expected by increasing the coupling strength from the sensing gate.

One of the unique features of the proposed ion sensor is its adjustable sensitivity and sensing range. The measured result of pH 3–9 is shown in Figure 8, where different sensitivities can be achieved by designing the contact length on the SG ($L_{1}$). For instance, decreasing $L_{1}$ leads to lower $\alpha_{\mathrm{SG}}$ and weaker coupling from the sense gate, stronger coupling from the read gate. Consequently, an output response having a wider sensing range with lower sensitivities is demonstrated. In contrast, increasing $L_{1}$ promotes the coupling strength from the sense gate, while reducing that from the read gate. Thus, the circuit exhibits a narrower sensing range with increased sensitivities.

### 3.2. Calibration Operation Scheme and Performance

**Figure 9:**
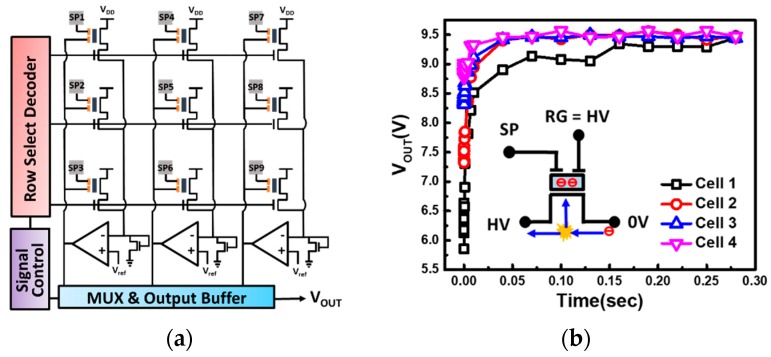
(**a**) Proposed pH sensing micro-array for obtaining ion distribution in liquid samples. (**b**) Channel hot electron injection is used for calibration, by which background offsets from different cells can be eliminated by designing the corresponding calibration time.

To provide precise sensor readings, it is critical that every sensor shows an identical response. However, due to process variation, the ion sensors are expected to be subject to large device-to-device differences, which can be even more significant as the scaling of device’s dimensions becomes aggressive. When incorporating a discrete sensor into a smart system, these inherent offsets are often addressed by calibration schemes in application software [27]. On the other hand, if one wishes to construct a sensing array (see Figure 9a) for the detection of pH level distribution, the in-cell self-calibration scheme becomes important to high fixed pattern noises, disturbing sensor readings. 

Here, a special calibration scheme which cancels the offsets in each sensor by performing the channel hot electron injection (CHEI) on this sensor device during testing phase is proposed for the first time. The operation condition and mechanism for electron injection are illustrated by the inset in Figure 9b. With high voltage applied on both the drain and RG terminal, channel carriers will be able to gain high enough energy for hot carrier injection into the FG, subsequently, correcting the offsets from each cell by adjusting its threshold voltage level. As shown in Figure 9b, cells with different initial output voltages can be trimmed effectively and self-converge to a saturated level. To initialize an array for sensing, all cells will go through the proposed calibration operation to provide a more uniform sensing response from each detector.

**Figure 10:**
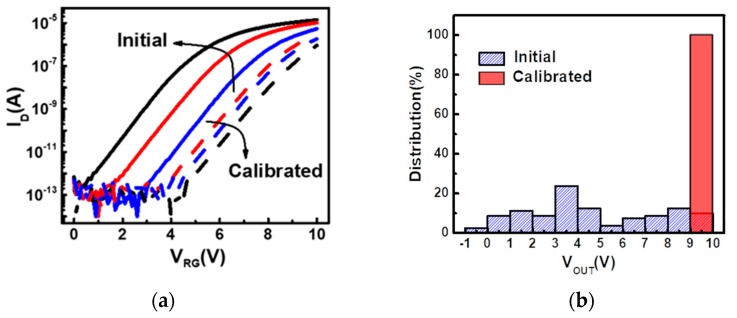
Comparisons of the (**a**) Current-Voltage (IV) characteristics of three different detectors before and after calibration. (**b**) The output voltage distributions of 80 initial and calibrated cells are summarized, showing significant tightening of characteristics in the calibrated group.

Here, we further compare the I-V characteristics before and after the calibration operation in Figure 10a. As revealed by the measured data, after electron injection, their transfer curves shift to the right, as expected. Resulting from the self-balanced characteristics, the device with lower threshold voltage will be programmed at a faster speed, and vice versa, and the output voltage distribution of 80 cells converges to a fair tight distribution after calibration, as shown in Figure 10b.

### 3.3. Self-Balanced Readout Circuit 

**Figure 11:**
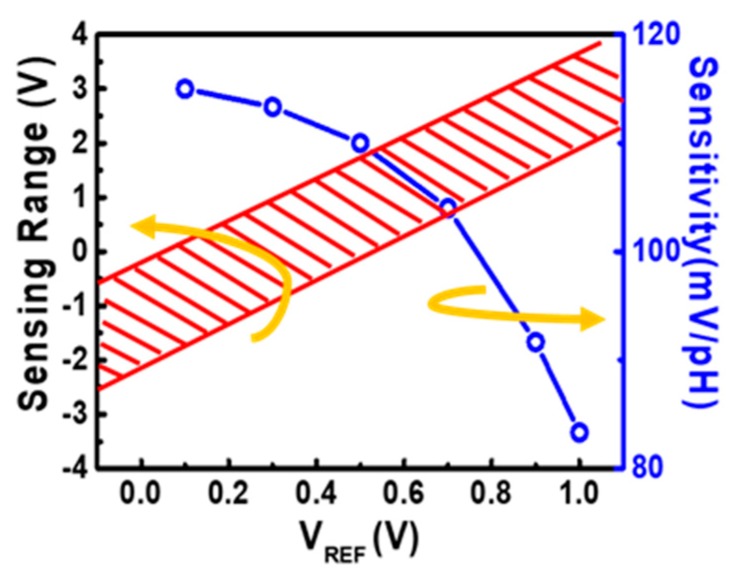
Sensing range of the sensor can be dynamically adjusted in accordance to the sample conditions by reference voltage levels for best sensor readout, while the sensitivities of the device change slightly when the reference potential changes.

As discussion in Section 3.1, the sensitivity of the proposed ion sensor can be adjusted by changing the corresponding contact length in the lateral coupling structure. However, once the device is made, its sensitivity is fixed. To provide a dynamic sensor for smart sensing, namely, adaptive toward environment changes, adjustable sensing response is a must. In the self-balanced readout circuit, the sensing range as well as sensitivities are both found to be modifiable by $V_{\mathrm{REF}}$. As shown in Figure 11 by simulation, as the $V_{\mathrm{REF}}$ increases, the sensing range will be shifted to a higher voltage range. Depending on the sensing application, its sensitivity on the other hand decreases slightly. The maximum sensitivity of this novel ion sensor is 115 mV/pH, which is amplified by the coupling ratios between SG and RG to FG. Additionally, the sensitivity of the proposed ion sensor can be described by the following equations [28,29]:

(11) $$V_{\mathrm{FG}}=\alpha_{\mathrm{SG}}\times \Psi_{0}+\alpha_{\mathrm{RG}}\times V_{\mathrm{RG}}+\frac{Q}{C_{\mathrm{total},\;\mathrm{FG}}},$$

(12) $$\frac{\Delta V_{\mathrm{OUT}}}{\Delta \mathrm{pH}}=\frac{\Delta V_{\mathrm{RG}}}{\Delta \mathrm{pH}}=\frac{\alpha_{\mathrm{SG}}}{\alpha_{\mathrm{RG}}}\left(-\frac{\Delta \Psi_{0}}{\Delta \mathrm{pH}}\right)\;\left(\frac{\mathrm{mV}}{\mathrm{pH}}\right),$$

where Q is the total charge stored in the FG, $\Psi_{0}$ is the interface potential, and V_FG_ is the FG potential fixed by the negative feedback readout circuit.

The major contributor of the intrinsic noise of operational amplifier is the thermal noise of the resistor. According to previous studies [30,31,32], such noise will be lower under low bandwidth operation. Therefore, the noise from the feedback operational amplifier can be found negligible compared to the noise from the testing environment.

**Figure 12:**
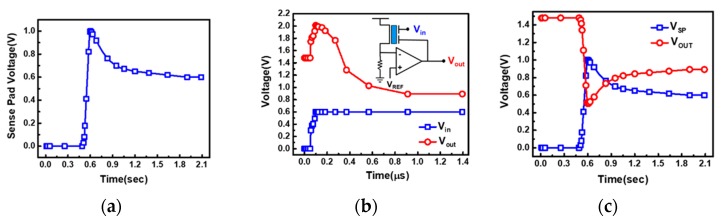
Real-time readout response of that (**a**) measured on sense pad, of the (**b**) readout circuit by simulation and (**c**) that combines both transient responses for the sense pad and readout circuit. The response time of the sense pad is found to be the bottleneck for sensor’s response time.

The response time of this readout scheme is further shown in Figure 12. The measured real-time response of SP shown in Figure 12a, suggesting that it required approximately 0.5 s for the SP voltage to reach its steady-state responding to a change of pH level. On the other hand, the simulated response time of the readout circuit is found to stabilize within one microsecond, as shown in Figure 12b. As the combined data revealed in Figure 12c, the total response time will be limited by the SP. It is expected that the response time of this detector can be further reduced by optimizing the sensing pad and the coating layer material or using iontophoresis as the H^+^ introduction, for high speed sensing applications such as rapid DNA sequencing in the future [33]. 

### 3.4. Multiple Ion/pH Sensing Application 

**Figure 13:**
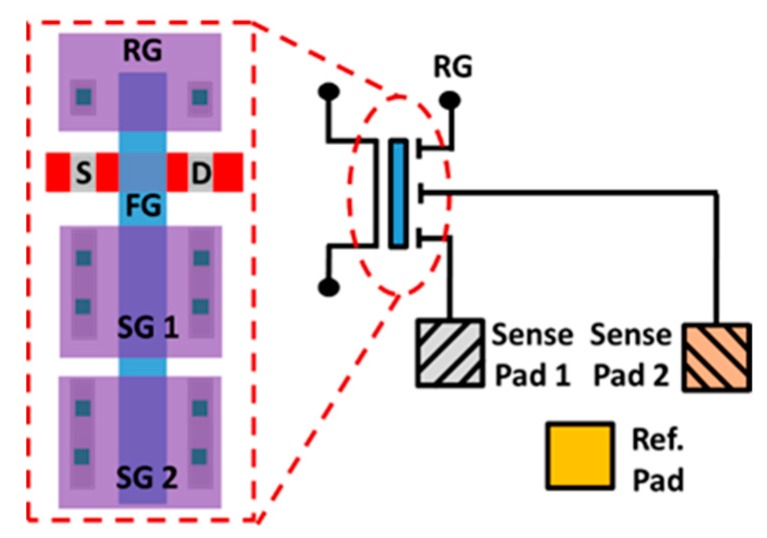
Schematic and layout of the proposed multi-ion/pH sensing device, with multiple sense pads coupled to the floating metal gate resulting in superposition results.

**Figure 14:**
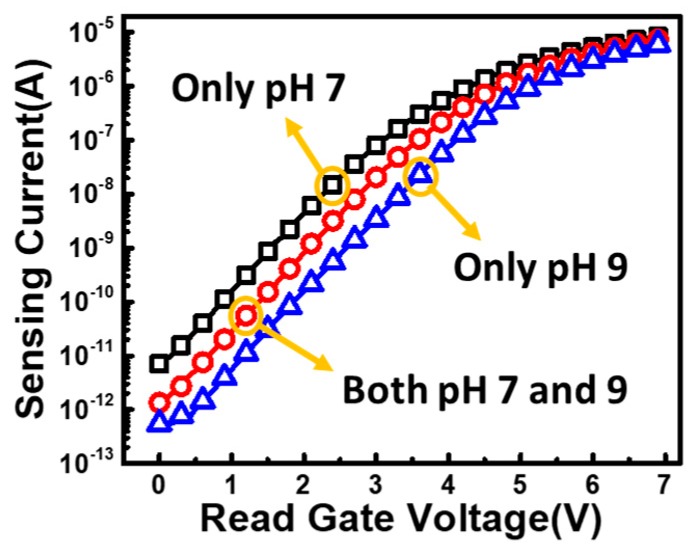
Measurement result of the superposition effect on the multi-pH sensing operation.

By adding another SG, the proposed ion sensor can also be used to detect multiple kinds of ions and pH levels as illustrated in Figure 13. To achieve multiple ion sensing in one single detector, different materials which are selectively sensitive to different ions can be coated on the top of SP1 and SP2, respectively. Due to the unique lateral coupling structure, the resulting response will be an “AND” of the signals for SP1 and SP2. Here, a demonstration of sensing the response of samples with different pH levels are shown. In Figure 14, a liquid sample of pH = 7 is placed on SP1, while another sample of pH = 9 is dropped on SP2. Data shows that superposition response of the two samples are obtained. Moreover, different weight can be given to different SP by drawing different contact lengths in the coupling structure from each gate to FG. The above feature allows this novel ion sensor to provide more flexible sensing results to meet the need for a variety of applications [34,35].

The superposition response can be used to detect, for example, saltiness. Na^+^ is the main contributor to taste of saltiness, while, K^+^ and other alkali metal ions may cause similar responses on the taste buds [36,37]. The readings can then directly reflect the summation of different ions present in a sample.

## 4. Conclusions

As listed in Table 1, compared to the conventional ISFET and EGFET, the proposed ion sensor in this work is CMOS compatible, meaning that no extra mask is needed, so the cost will be lower for mass production. The sensitivity of this device can reach to 115 mV/pH through an internal amplification effect by the coupling structure. Furthermore, the sensitivity and sensing range can be optimized by designing the coupling ratio for each input gate. The reference voltage provides a flexible tool to adjust the sensing range of the sense pad for adaptive applications. Transient response time and SNR was discussed in detail, which can be directions for further performance improvement in the future. Additionally, a calibration scheme is proposed and demonstrated to provide a robust offset canceling method when a micro-detector array is in need. Lastly, this ion sensor was demonstrated to the sensing of multiple ions in a single device, showing potential for the capacity for extended applications, like biomedical diagnostics, soil-quality testing and so on [34,35]. 

## Figures and Tables

**Table 1:** Performance comparison between other reported ion sensors and this work.

	Ref. [26]	Ref. [38]	Ref. [39]	This Work
Type	ISFET	ISFET	EGFET	FG FinFET
Technology	Si-Nanonet	0.35 μm CMOS	Glass Substrate	16 nm FinFET
Sensitivity(mV/pH)	48.1	39.6	57.9	115
SNR (dB)	35.31	23.01	N/A	30.14
Multi-ion/pH	No	No	No	Yes
